# No Significant Evidence of Cognitive Biases for Emotional Stimuli in Children At-Risk of Developing Anxiety Disorders

**DOI:** 10.1007/s10802-015-0122-8

**Published:** 2016-01-08

**Authors:** Donna L. Ewing, Suzanne Dash, Ellen J. Thompson, Cassie M. Hazell, Zoe Hughes, Kathryn J. Lester, Sam Cartwright-Hatton

**Affiliations:** Trafford Centre, Brighton and Sussex Medical School, University of Sussex, Falmer, BN1 9RY UK

**Keywords:** Anxiety, Cognitive bias, At-risk children, Emotion recognition, Ambiguous situations

## Abstract

This paper explores whether the increased vulnerability of children of anxious parents to develop anxiety disorders may be partially explained by these children having increased cognitive biases towards threat compared with children of non-anxious parents. Parents completed questionnaires about their child’s anxiety symptoms. Children aged 5–9 (*n* = 85) participated in two cognitive bias tasks: 1) an emotion recognition task, and 2) an ambiguous situations questionnaire. For the emotion recognition task, there were no significant differences between at-risk children and children of non-anxious parents in their cognitive bias scores for reaction times or for accuracy in identifying angry or happy facial expressions. In addition, there were no significant differences between at-risk children and children of non-anxious parents in the number of threat interpretations made for the ambiguous situations questionnaire. It is possible that these cognitive biases only become present subsequent to the development of an anxiety disorder, or only in older at-risk children.

Children with anxiety disorders show cognitive biases towards threat-relevant stimuli, such as attentional biases (Waters et al. 2010), emotion recognition biases (Simonian et al. 2001), and interpretation biases (Bögels and Zigterman 2000). However, there is little research that has explored whether these biases are present in children who are at-risk of developing anxiety disorders because one or both of their parents have an anxiety disorder. It is well established that anxiety runs in families (Eley et al. 2015; Turner et al. 1987), with evidence to suggest that children of anxious parents are up to seven times more likely to also be diagnosed with an anxiety disorder themselves (Turner et al. 1987). We wanted to explore whether children at-risk of developing anxiety already show cognitive biases towards threat. Alternatively, the absence of cognitive biases in at-risk children may suggest that these biases become present only as a result of the developing anxiety disorder. The current paper offers a preliminary exploration of this question by considering whether threat-relevant emotion recognition and interpretation biases are present in children at-risk of developing anxiety disorders.

## Emotion Recognition

Anxious adults demonstrate emotion recognition biases towards threat, with high trait anxious adults showing vigilance in identifying fearful emotional expressions compared with low trait anxious adults (Surcinelli et al. 2006). Similarly, socially anxious adults required fewer facial features (that is, just eyebrows rather than eyebrows and mouth) to interpret the facial expression as threatening, compared with non-anxious adults (Coles et al. 2008).

As in the adult literature, a range of childhood psychiatric disorders are associated with differences and difficulties in facial emotion recognition, including schizophrenia, mood disorders, anxiety disorders, and attentional-deficit hyperactivity disorder (Collin et al. 2013). Children with anxiety disorders have been found to show an attentional bias towards threatening emotional face stimuli. For instance, more severely anxious children were found to have greater attentional bias towards angry faces when paired with neutral faces, compared with less anxious and non-anxious children (Krain Roy et al. 2008; Waters et al. 2008b, 2010), and anxious youth made faster eye fixations to angry than neutral faces compared with non-anxious youth (Shechner et al. 2013).

In addition, children with social phobia have greater difficulties in accurate emotion recognition, making, for example, a greater number of errors in recognising happy, sad and disgust emotions in pictures of adults when compared to children without social phobia (Simonian et al. 2001). However, accuracy in emotion recognition may be improved when children with symptoms of social anxiety are presented with child rather than adult faces (Ale et al. 2010), and both accuracy and reaction times for identifying emotional expressions have been found to improve with age (Broeren et al. 2011). On the other hand, other research suggests that social anxiety may enhance children’s emotion recognition abilities, with one study showing that children who had greater social anxiety symptoms were better at emotion recognition (Ale et al. 2010). Similarly, research using a sample of children with a range of anxiety disorders found that anxious children were equally able to identify emotional expressions in child faces when compared with healthy controls (McClure et al. 2003).

Although there is evidence that both anxious adults and children show emotion recognition biases towards threat, little is known about the pattern of biases in children who are at risk of anxiety as a result of their parent’s anxiety disorder.

## Interpretation Bias

Ambiguous stimuli are often employed to measure threat interpretation biases in children with anxiety, for example, ambiguous situations or scenarios (e.g., Bögels and Zigterman 2000; Muris et al. 2000b; Waters et al. 2008a), or homophone words (Taghavi et al. 2000). Findings from these studies suggest that anxious children show more threat interpretation biases towards, and more negative emotions about, the ambiguous stimuli compared with non-anxious children. For instance, when asked to incorporate ambiguous homograph words (for example, die/dye) into a written sentence, anxious children were more likely to interpret the words as threat words than non-anxious children (Taghavi et al. 2000). Similarly, when presented with an ambiguous situation or scenario, anxious children were more likely to place a threat interpretation on the situation compared with non-anxious children (Bögels and Zigterman 2000), particularly when the situation was more personally salient to the child (Micco and Ehrenreich 2008). Within interpretation bias tasks, anxiety was correlated with a higher frequency of reports of threat and ratings of how threatening a story was, more threat interpretations, and greater negative feelings and cognitions (Muris et al. 2000a). Findings from longitudinal research suggest that the association between threat interpretation biases and childhood anxiety increased across time, and was bidirectional, in that threat interpretation bias was predicted by anxiety symptoms, while anticipated distress for the ambiguous situations predicted change in anxiety (Creswell and O’Connor 2011).

Various factors have been shown to influence children’s tendency towards threat interpretation biases, including external and internal influences, such as: parenting (Creswell et al. 2005), positive or negative feedback (Lester et al. 2011a, b), or bodily sensations (Muris et al. 2010). In particular, significant associations were found between mother and child threat interpretation biases (Creswell et al. 2005), and children’s interpretation biases partially mediated the relationship between an over-controlling parenting style and child anxiety (Affrunti and Ginsburg 2012). In addition, children of parents who encouraged them to seek out negative information about novel animals were found to show an increase in their search for negative information and a decrease in their search for positive information, while children of parents who encouraged them to seek out positive information showed a reduction in the number of negative questions the children asked about the animals, and an increase in their search for positive information (Remmerswaal et al. 2015) suggesting that parents were able to influence children’s cognitive biases. Therefore, it is possible that having an anxious parent could result in children showing signs of threat interpretation biases, even if the child is not currently anxious themselves. This may particularly be the case if the parent shows biases about child-relevant threats (Lester et al. 2009, 2012).

However, a study that has explored such biases in at-risk children (aged 7–12) did not find a significant difference between the at-risk children and children of non-anxious parents (Waters et al. 2008a). Waters et al. (2008a) suggest that this may be because children of anxious parents are no more vulnerable to developing threat interpretation biases than children of non-anxious parents, or because the task was not sensitive enough to detect bias in this sample. However, these findings were also based on underpowered analyses. Other research has found that, compared with children (aged 8–15 years) of non-anxious parents, having a parent with panic disorder increases a child’s vulnerability to provide panic interpretations for ambiguous situations after being primed about panic attack symptoms (Schneider et al. 2002). However, this study did not consider whether these children showed this vulnerability prior to being primed. Other research found that children (aged 7–14) of mothers with elevated symptoms of *both* depression and agoraphobia, or *both* depression and interoceptive fears (that is, fears of automatic bodily sensations), showed increased interpretation biases towards threat for separation anxiety concerns, compared to children of mothers with typical levels of depression only (Perez-Olivas et al. 2011).

## Current Study

The current study explores whether children at-risk of developing anxiety disorders (because their parent has a clinical diagnosis of an anxiety disorder) are more likely to show emotion recognition biases and threat interpretation biases compared with children of non-anxious parents.

## Method

### Participants

The initial sample included 95 children (46 children of anxious parents (at-risk children) and 49 children of non-anxious parents) aged 5–9. Where parents had more than one child between ages 5–9 years, parents chose which child to consent. Anxious parents were referred from local mental health services or were self-referred. Of the anxious parents referred from local mental health services, a further 29 did not participate for a number of reasons: contact couldn’t be made with the participant (*n* = 13), the participant felt unable to commit to participation due to difficulties with their anxiety (*n* = 5), the participant was too busy to participate (*n* = 1), the participant didn’t want their child to be involved (*n* = 1), the participant declined without giving a reason (*n* = 1), or the participant did not attend (*n* = 5).

To be included in the anxious sample, parents had to achieve diagnosis of at least one anxiety disorder diagnosis according to meeting criteria for an anxiety disorder on the PDSQ (see below) and scoring at or above the suggested clinical cut-off on the Spielberger trait anxiety scale (see below). For the majority of participants, this diagnosis was later confirmed using the ADIS (see below). However, for a minority (seven parents) the ADIS could not be completed as contact could not be made following participation in the study. Independent samples t-tests confirmed there were no significant differences between these seven parents (and their children) and the parents who completed the ADIS (and their children) in age or gender, in parent-rated anxiety scores, or in child cognitive biases scores (*p* > 0.05).

Parents were included in the non-anxious sample if they scored below suggested clinical cut-off on the Spielberger trait anxiety scale (see below) *and* did not meet criteria for any anxiety diagnosis on the PDSQ (see below). Two parents were excluded on this basis.

Therefore, a total sample of 85 children (37 % female) aged 5–9 years (mean = 6.92 years, *SD* = 1.30) and their parents (either mothers or fathers), remained for analysis. The majority of the children were of White British ethnicity (89.3 %), with other ethnic groups including White Other (2.4 %), Mixed White and Asian (3.6 %), Mixed White and Black (1.2 %), Mixed Other (2.4 %), and Other (1.2 %), which, with the exception of an under-representation of Black/Black British and Asian/Asian British ethnic groups, is comparable to that of the ethnicity across the population of England and Wales (ONS 2011). Approximately half of the child participants (*n* = 44; 36 % female; mean age = 7.07 years, *SD* = 1.30) were children of parents with clinical anxiety disorder diagnoses (86 % mothers; mean age = 40.52, *SD* = 5.95; mean clinician severity rating (CSR) = 6.22; SD = 1.30, range = 4–8) and approximately half (*n* = 41; 37 % female; mean age = 6.76 years, *SD* = 1.30) were children of non-anxious parents (90 % mothers; mean age = 40.82, *SD* = 5.12). Parent anxiety disorder diagnoses included: generalised anxiety disorder (*n* = 26); social anxiety disorder (*n* = 22); panic disorder with agoraphobia (*n* = 13); panic disorder without agoraphobia (*n* = 3); agoraphobia with no history of panic disorder (*n* = 1); obsessive compulsive disorder (*n* = 10); specific phobia (*n* = 10); health anxiety (*n* = 5) and posttraumatic stress disorder (*n* = 5). Parents and children participated in a number of experimental tasks. This paper reports on tasks completed by children only.

All parents included in the study had a child aged 5–9 years, spoke English to a good standard, and neither parent nor the child had major developmental or intellectual disabilities. Exclusion criteria for clinical participants included parents who did not meet diagnostic criteria for an anxiety disorder, lacked the capacity to consent to participation according to referrers’ opinions, and whose needs were inappropriate for a group-based intervention (as required for a subsequent group-based intervention study). This included a current active psychosis, severe depression, current manic state or certain Axis II conditions. Non-clinical participants were recruited through adverts placed in local newspapers, parenting magazines, and the study website, and through contacting a database of parents who had previously taken part in other developmental studies at the University.

### Measures

#### Demographics

Background information was collected on parent and child, including date of birth, gender, and ethnicity.

#### Anxiety Disorders Interview Schedule (ADIS) - Adult Version (Brown et al. 1994)

The ADIS was completed by a trained clinical research officer, with adult clinical participants only, in order to confirm their anxiety diagnosis. The following sections were used in the current study: panic disorder, agoraphobia, social phobia, generalized anxiety disorder, obsessive compulsive disorder, specific phobia, posttraumatic stress disorder, and hypochondriasis. Sections on major depressive disorder, dysthymic disorder, mania/cyclothymia, somatization disorder, mixed anxiety/depressive disorder, alcohol abuse/dependence, substance abuse/dependence, and non-organic psychosis were excluded from the interview.

#### Psychiatric Diagnostic Screening Questionnaire (PDSQ; Zimmerman and Mattia 2001)

The PDSQ is a self-rated diagnostic questionnaire on a 2-point scale (*yes*, *no*). The PDSQ was adapted for the current study, removing the sections for major depressive disorder, bulimia/binge-eating disorder, psychosis, alcohol abuse/dependence, drug abuse/dependence, and somatization disorder, as only sections relating to anxiety were required. A total of 57 items remained, covering symptoms of obsessive compulsive disorder (OCD), panic disorder (PD), agoraphobia, social phobia (SP), generalised anxiety disorder (GAD), health anxiety, and post-traumatic stress disorder (PTSD). The PTSD section was moved to the end of the questionnaire as, after removing non-anxiety specific items, this section came first, which was not considered ideal due to the potentially distressing nature of the questions. All other sections remained in the same order as the original version. Convergent and discriminant validity of the PDSQ has been demonstrated, and the instrument has a sensitivity of 87 % & negative predictive power of 97 % (Zimmerman and Mattia 2001). For the current sample, the PDSQ was found to have good-to-excellent internal consistency across the subscales (α = 0.76–0.92).

#### Spielberger State-Trait Anxiety Inventory (STAI; Spielberger et al. 1970)

The STAI is a self-rated questionnaire measuring state and trait anxiety. The STAI has 40 items (20 measure state anxiety; 20 measure trait anxiety) rated using a 4-point scale (*almost never*, *sometimes*, *often*, *almost always*). The Trait Anxiety scale of the STAI has good test-retest reliability, with correlations ranging from 0.73 to 0.86 (Spielberger et al. 1970). High internal consistency has been found for both the Trait Anxiety scale (α = 0.89–0.91) and for the State Anxiety scale (α = 0.86–0.95). The construct and concurrent validity of the STAI has also been demonstrated (Spielberger et al. 1970). For the current sample, high internal consistency was found for both the Trait Anxiety scale (α = 0.96) and for the State Anxiety scale (α = 0.92).

#### Spence Child Anxiety Scale (Parent Rating) (SCAS; Spence 1998)

The SCAS is a parent-rated questionnaire for an overall measure of child anxiety, including measures of panic and agoraphobia, separation anxiety, physical injury fears, social phobia, obsessive compulsive disorder, and generalised anxiety disorder. The SCAS has 38 items rated on a 4-point scale with scores of 0–3 given to responses of *never*, *sometimes*, *often*, and *always*, respectively. This measure uses a clinical cut off of 31.4 for boys, and 33 for girls. This scale has good- to excellent internal consistency, with Cronbach alphas ranging from 0.61 to 0.92 across the subscales, and has good validity, indicated by strong correlations (*r* = 0.55–0.59) between this scale and the internalising subscale of the Child Behaviour Checklist (Nauta et al. 2004). In addition, the SCAS shows good discriminant validity in terms of identifying children who reach clinical cut-offs for anxiety diagnoses (Nauta et al. 2004). For the current sample, Cronbach alphas ranged from 0.38 to 0.76 across the subscales.

#### The Ambiguous Situations Questionnaire (ASQ; Barrett et al. 1996; Creswell et al. 2005)

This questionnaire consisted of 12 ambiguous sentences that could be interpreted in either a threatening or non-threatening way, with six social threat sentences and six physical threat sentences (e.g., “you are on your way to your friend’s house when a big dog comes up to you”). The questions were displayed in E-Prime (version 2.0) on a Dell laptop. Children were introduced to the task and were told “Here are some situations that you might find yourself in. You might have been in some of these situations before. For others you might have to imagine what it would be like to be in that situation. The important thing is that you say what you would really think if it happened to you and what you would really do”. For each trial, children were presented with the ambiguous situation on the computer screen, which was read out to the children. The children were then asked what they thought was happening. Children responded with freely spoken interpretations of the ambiguous situations, which were recorded using a microphone attached to the computer. Children were then given two forced choice interpretations of the sentence and were asked “Which of these do you think is most likely?” Children selected one of these using the appropriately marked key on the keyboard. The spoken and forced-choice responses were coded as threat or non-threat responses. Responses that were rated as either positive or neutral outcomes to the ambiguous situation were coded as non-threat responses (for example, “the dog has come to play with me”), while negative outcomes to the situation were coded as threat responses (for example, “the dog will bite me”). Non-responses or responses of “I don’t know” were coded as missing data. A social threat score was calculated based on the total number of social threats identified. A physical threat score was calculated based on the total number of physical threats identified. A total threat score was calculated based on the total number of threats identified across all of the questions. Free verbal responses were coded by the first author and social and physical threat responses for 22 % of the participants (*n* = 11) were second-rated by a research assistant. Substantial inter-rater agreement was found for the coding of the child verbal responses, with Kappa scores ranging from 0.62 to 1.00 across the questions. The scores from the first rater were used for analysis.

#### Emotion Recognition Task (Broeren et al. 2011; Brown et al. 2014)

This task was presented in Eprime (version 2.0) on a Dell laptop. Twenty models (50 % female) with open and closed mouth facial expressions (neutral, happy and angry) were taken from the NimStim set of facial expressions (Tottenham et al. 2009). Each neutral expression was morphed in 75 increments of increasing emotional intensity for angry and happy facial expressions for each model, resulting in a total of 40 dynamic face morphs for 40 trials. Children were shown trials of short digital movies (10 s) in which the neutral face gradually changed into a full blown happy or angry emotional expression. Each trial was preceded by a fixation cross displayed for 1000-2000 ms. Children indicated (as quickly and as accurately as possible) which emotional expression was being displayed by pressing an appropriate key on a serial response box as soon as they recognised the emotion. The trial concluded once the child responded, and the next trial began. This was repeated until all 40 trials were complete. Reaction times and accuracy of the children’s responses were recorded.

### Procedures

Ethical approval was received from the authors’ university and the NHS Research Ethics Service. The study was explained to the parent and the child, and each gave consent for the child to take part. Parents and children completed the questionnaires prior to the children completing two computer-based tasks. The first task involved an emotion recognition task, and the second involved an interpretation bias task (each as described above).

### Data Preparation

The emotion recognition data were available for 85 children. The practice data of the morph task was scrutinised for errors, and data for children with more than 2 errors during this practice phase were excluded, as suggested by Broeren et al. (2011), as these children may not have fully understood or engaged with the task (*n* = 5). After removing these data, there remained a few outlier scores for the number of errors made in the main task. A decision was made to exclude the data of children that fell outside of 2.5 standard deviations of the mean number of errors made (*n* = 3). After removing these outliers (at-risk children: *n* = 4; control group children: *n* = 4), morph data for the main task was available for 77 children, with a mean number of 2.01 errors (SD = 2.56).

The forced choice interpretation bias data were available for 76 children. Owing to instrument failure, verbal response data was available for 48 of the 76 children. Participants with and without verbal response data were compared, and there were no significant differences between groups in gender, ethnicity, age, or SCAS anxiety scores (*p* > 0.05). However, children with missing data were significantly more likely to be children of non-clinically anxious parents, *t*(74) = 5.12, *p* < 0.001. Correlational analyses were conducted between the forced choice and free verbal responses to the ASQ. There were no significant correlations between the forced choice and free verbal responses for the total number of threats (*r* = 0.22, *p* = 0.14), and so a decision was made to consider the forced choice and free verbal responses to this task separately. It is possible that children respond differently using forced choice and free verbal responses, with children’s verbal responses potentially more representative of their initial reaction to the situations, while forced choice responses may give children a chance to consider alternative outcomes.

### Data Analysis Plan

To achieve 80 % power to detect a medium effect size (*d*) of 0.6 assuming an alpha of 0.05, a sample of 72 participants was required (with 36 participants in the two samples: at-risk children and children of non-anxious parents). Therefore, 86 % power was achieved for the emotion recognition task based on the 85 participants recruited (44 at-risk children and 41 children of non-anxious parents) and 83 % power was achieved for the forced-choice section of the interpretation bias task, based on the 76 participants recruited (39 at-risk children and 37 children of non-anxious parents). Due to instrument failure, data for the free verbal responses on the interpretation bias task was available for 34 at-risk children and 14 children of non-anxious parents, and so 59 % power was achieved.

Independent samples t-tests were conducted to compare the sample of children at-risk of developing anxiety with the sample of children of non-anxious parents in terms of demographics, anxiety scores, cognitive bias scores, and interpretation bias scores. Linear hierarchical regressions were used to explore whether children’s at-risk status, anxiety symptoms, age, and interaction between at-risk status and age predict cognitive biases for the emotion recognition task and/or interpretation biases for the ambiguous situations task.

## Results

### Demographic Differences Between the At-Risk and Non-Anxious Samples

Independent samples t-tests were conducted to test for demographic differences between the sample of children at risk of developing an anxiety disorder and the sample of children of non-anxious parents. There were no significant differences between the samples in terms of age, *t*(83) = 1.11, *p* = 0.27, or gender, *t*(83) = 0.02, *p* = 0.98.

### Anxiety Symptoms

SCAS anxiety scores were relatively low across the sample, with the mean SCAS score falling well below the clinical cut-off (mean = 19.82; suggested clinical cut-off = 31.4 for boys, and 33 for girls). There was a significant difference in SCAS anxiety scores between the sample of children at-risk of developing an anxiety disorder and children of non-anxious parents, *t*(72) = 3.62, *p* < 0.001, with at-risk children scoring significantly higher (but still below clinical cut-off) than children of non-anxious parents (see Table 1).

**Table 1:** Mean and standard deviations for children’s SCAS anxiety scores

	Mean SCAS score (SD)
At-risk children (*n* = 39)	23.36 (9.64)
Children of non-anxious parents (*n* = 35)	15.89 (7.90)

### Emotion Recognition

Cognitive bias scores were calculated by subtracting the reaction time for recognising happy faces from the reaction time for recognising angry faces on the face morph task (see Table 2), with positive scores reflecting a bias towards happy faces (that is, a faster reaction time or greater accuracy to detect happy emotional expressions compared with angry emotional expressions). Independent samples t-tests were conducted to consider whether there were any significant differences in cognitive bias scores for children at-risk of developing an anxiety disorder compared with children of non-anxious parents. The results of the t-tests suggested that there was no significant difference between at-risk children and children of non-anxious parents in their cognitive bias scores for reaction times, *t*(75) = 0.88, *p* = 0.38, *d* = −0.20, or for accuracy, *t*(75) = 1.08, *p* = 0.28, *d* = −0.24. No significant correlations were found between children’s SCAS scores and their cognitive bias scores for reaction time, *r* = −0.09, *p* = 0.49, or for accuracy, *r* = 0.07, *p* = 0.56.

**Table 2:** Means and standard deviations for reaction times and accuracy during the emotion recognition task

	Reaction time (ms) Mean (SD)	Accuracy (number of errors) Mean (SD)
Angry stimuli		
All children (*n* = 77) At-risk children (*n* = 40) Children of non-anxious parents (*n* = 37)	5584.44 (1381.85) 5528.98 (1542.81) 5644.41 (1202.27)	0.83 (1.45) 0.68 (0.97) 1.00 (1.84)
Happy stimuli		
All children (*n* = 77) At-risk children (*n* = 40) Children of non-anxious parents (*n* = 37)	5183.42 (1398.27) 5049.91 (1578.80) 5327.76 (1177.29)	1.18 (1.71) 1.25 (1.79) 1.11 (1.65)
Cognitive bias score		
All children (*n* = 77) At-risk children (*n* = 40) Children of non-anxious parents (*n* = 37)	401.02 (805.07) 479.07 (801.66) 316.65 (811.17)	−0.35 (1.89) −0.58 (1.36) −0.11 (2.33)

Linear hierarchical regressions were conducted to consider whether children’s at-risk status, SCAS symptoms of anxiety (high levels of which may indicate early signs of a developing anxiety disorder), and age predict their cognitive bias scores for reaction times and accuracy on the emotion recognition task, with at-risk status entered in the first model, SCAS anxiety scores entered in the second model, child age entered in the third model, and the interaction between at-risk status and child age entered in the fourth model. These variables were not found to predict children’s cognitive bias scores for reaction time, Model 1: *F*(1, 64) = 0.38, *p* = 0.54, *r*^2^ = 0.01; Model 2: *F*(2, 63) = 0.70, *p* = 0.50, *r*^2^ = 0.02; Model 3: *F*(3, 62) = 0.51, *p* = 0.68, *r*^2^ = 0.02; Model 4: *F*(4, 61) = 1.58, *p* = 0.19, *r*^2^ = 0.09, or for accuracy, Model 1: *F*(1, 64) = 0.77, *p* = . 39, *r*^2^ = 0.01; Model 2: *F*(2, 63) = 0.42, *p* = 0.66, *r*^2^ = 0.01; Model 3: *F*(3, 62) = 0.28, *p* = 0.84, *r*^2^ = 0.01; Model 4: *F*(4, 61) = 0.79, *p* = 0.54, *r*^2^ = 0.05.

### Interpretation Bias

To explore whether there were any differences in the number of *forced-choice* threats selected in the ambiguous scenarios between children at-risk of anxiety and children of non-anxious parents, independent samples t-tests were conducted. No significant differences were found between at-risk children and children of non-anxious parents in terms of the number of forced-choice social threats, *t*(74) = 0.69, *p* = 0.49, *d* = 0.16, physical threats, *t*(74) = 0.41, *p* = 0.68, *d* = 0.09, or total threats, t(74) = 0.67, *p* = 0.51, *d* = 0.15 (see Table 3 for means and standard deviations). No significant correlations were found between SCAS anxiety scores and the number of social threats, *r* = 0.01, *p* = 0.96, physical threats, *r* = 0.05, *p* = 0.68, or total threats identified, *r* = 0.04, *p* = 0.77.

**Table 3:** Means and standard deviations for the number of threats (both forced choice and free verbal response) identified in the ambiguous situations questionnaire

	Forced choice		Free verbal response	
Threat type	At-risk sample (*n* = 39) Mean (SD)	Children of non-anxious parents (*n* = 37) Mean (SD)	At-risk sample (*n* = 34) Mean (SD)	Children of non-anxious parents (*n* = 14) Mean (SD)
Social threats	2.44 (1.27)	2.22 (1.49)	3.06 (1.30)	3.50 (1.22)
Physical threats	2.51 (1.45)	2.38 (1.40)	2.21 (1.43)	1.50 (1.02)
Total threats	4.94 (2.43)	4.59 (2.20)	5.21 (2.14)	5.00 (1.84)

To explore whether there were any differences in the number of *free verbal response* threat interpretations in the ambiguous scenarios between children at-risk of anxiety and children of non-anxious parents, independent samples t-tests were conducted. No significant differences were found between at-risk children and children of non-anxious parents in terms of the number of free response social threat, *t*( 46) = −1.09, *p* = 0.28, *d* = −0.35, physical threat, *t*(45) = 1.69, *p* = 0.10, *d* = 0.57, or total threat, *t*(46) = 0.31, *p* = 0.76, *d* = 0.10, interpretations made (see Table 3 for means and standard deviations). No significant correlations were found between SCAS anxiety scores and the number of physical threats, *r* = 0.08, *p* = 0.63, or total threats identified, *r* = −0.17, *p* = 0.28. A modest negative correlation was found between SCAS anxiety scores and the number of social threats, *r* = −0.37, *p* < 0.05.

Linear hierarchical regression analyses were conducted to consider whether children’s at-risk status, symptoms of anxiety, and age predict the number of forced-choice and free verbal response threat interpretations made, with at-risk status entered in the first model, SCAS anxiety scores entered in the second model, age entered in the third model, and the interaction between at-risk status and age entered in the fourth model. These variables were not found to predict children’s threat interpretation biases for *forced choice* social threat interpretations, Model 1: *F*(1, 62 = 0.65, *p* = 0.42, *r*^2^ = 0.01; Model 2: *F*(2, 61) = 0.34, *p* = 0.72, *r*^2^ = 0.01; Model 3: *F*(3, 60) = 2.26, *p* = 0.09, *r*^2^ = 0.10; Model 4: *F*(4, 59) = 1.78, *p* = 0.15, *r*^2^ = 0.11. Likewise, at-risk status and SCAS anxiety scores did not predict forced choice physical threat interpretations, Model 1: *F*(1, 62 = 0.02, *p* = 0.89, *r*^2^ < 0.01; Model 2: *F*(2, 61) = 0.06, *p* = 0.94, *r*^2^ = 0.00. Age predicted children’s threat interpretation bias scores for forced choice physical threat interpretations, Model 3: *F*(3, 60) = 3.66, *p* < 0.05, *r*^2^ = 0.16, with older children making fewer physical threat interpretations, β = −0.42, *p* < 0.01. Model 4 (with the interaction between at-risk status and age) did not significantly change R-squared for this analysis, Δ*r*^2^ = 0.001, *p* > 0.05.

At-risk status, SCAS anxiety symptoms, and age did not predict children’s threat interpretation biases for *free verbal response* physical threat interpretations, Model 1: F(1, 40) = 3.17, *p* = 0.08. *r*^2^ = 0.07; Model 2: *F*(2, 39) = 1.58, *p* = 0.22, *r*^2^ = 0.08; Model 3: *F*(3, 38) = 1.79, *p* = 0.17, *r*^2^ = 0.12; Model 4: *F*(4, 37) = 1.35, *p* = 0.27, *r*^2^ = 0.13. At-risk status, age, and the interaction between at-risk status and age did not significantly predict free verbal response social threat interpretations, Model 1: *F*(1, 41) = 1.12, *p* = 0.30, *r*^2^ = 0.03; Model 3: *F*(3, 39) = 2.30, *p* = 0.09, *r*^2^ = 0.15; Model 4: *F*(4, 38) = 1.89, *p* = 0.13, *r*^2^ = 0.17. However, including SCAS anxiety scores in the model accounted for 12 % of the variance in free verbal response social threat interpretations, Model 2: *F*(2, 40) = 3.48, *p* < 0.05, *r*^2^ = 0.15, with greater symptoms of anxiety predicting fewer social threat interpretations.

## Discussion

The findings of this paper suggest that 5–9 year old children who are at risk of anxiety disorders, as a result of a parental anxiety disorder diagnosis, are no more likely to display threat interpretation or emotion recognition biases than their low-risk peers.

The threat-interpretation findings support and replicate the findings of Waters et al. (2008a) who also found no differences in threat interpretation biases in children at-risk of developing anxiety and children of non-anxious parents. However, the current study used a larger sample than Waters et al. (2008a), meaning that the null results are less likely to have arisen due to lack of power. The findings also support those that found no significant differences between at-risk and low-risk children in their attentional bias towards emotional faces (Waters et al. 2015). Together, these findings suggest that emotion recognition and threat interpretation biases are not present to a greater than expected extent in children who are at-risk of developing anxiety disorders. Other research has suggested that children of parents with panic disorder show increased attentional biases towards threat compared to children of parents without psychiatric disorder (Mogg et al. 2012). However, that study employed children approaching and in adolescence (9–14 years), and parents who had a single anxiety diagnosis (panic disorder), which may account for the different outcome.

The inhibition model of anxiety acquisition proposes that biases towards threat are normative in early childhood, and that these biases only become inhibited during later development, except for in fearful children, where they are maintained (Kindt et al. 2000). Results of the interpretation bias task in the current study support this model, as age was found to negatively predict threat interpretation biases in relation to physical threats, with the identification of physical threats in ambiguous situations decreasing with age, irrespective of at-risk status. However, neither at-risk status, nor age, nor their interaction, were found to predict children’s cognitive biases in the emotion recognition task, which suggests that age does not predict all cognitive biases. Results from the emotion recognition task showed some evidence of children, irrespective of age, showing a cognitive bias towards happy stimuli, with children generally slower to identify the angry facial stimuli when compared to the happy facial stimuli. Therefore, results from this study suggest that biases towards threat stimuli may only appear once an anxiety disorder begins to develop. Our at-risk sample were a rather healthy group, with few signs of emerging anxiety disorders at this stage.

Despite its strengths, notably the use of a large, clinically at-risk sample of children, the current study was not without weaknesses. In particular, parents were free to choose which of their children they should bring into the laboratory. It is possible that parents (in both samples) will have opted only to bring children who they considered to be ‘robust’ and able to cope with the stresses of participation. If this is the case, genuine cognitive biases, that were present in their other children, will have been missed. In addition, we were only able to assess the anxiety status of the one attending parent of the ‘healthy’ sample. It is possible that some of the children in this group will have had another parent who did experience significant anxiety. Another limitation of this study is the lack of diagnostic information for the children included in this study, which means that it was not possible to consider whether children already affected by anxiety disorders demonstrated increased cognitive biases towards threat compared to children without anxiety disorders. However, given the relatively low anxiety scores based on the parent-rated SCAS questionnaire (with the mean well below clinical cut-off), it is unlikely that a sufficient proportion of the sample would have met diagnostic criteria for an anxiety disorder in order to run these analyses. Unfortunately, due to a technical difficulty, some free verbal response data from the interpretation bias task was lost. As a result, the analyses for this data were underpowered, and it is possible that the lack of significant results were due to Type II error. It would, therefore, be useful for further research to replicate this task using well-powered analyses.

More research with the children of clinically anxious parents is clearly needed. Research exploring similar biases using more sensitive methods may demonstrate differences in at-risk children that were not apparent here, or confirm that they are not present. Similarly, there are many other biases that could be present in children at risk of anxiety disorders, and these also need investigating.

## Conclusion

This paper addresses an important gap in current literature through the consideration of cognitive biases in children of anxious parents. The non-significant differences found in cognitive biases between at-risk children and children of parents without anxiety disorders suggest that young at-risk children are no more vigilant in detecting threat than children of non-anxious parents. This may suggest that having an anxious parent does not increase a child’s risk of having cognitive biases towards threat, at least at this stage of development. Therefore, the increased risk of anxiety in children of anxious parents does not appear to be explained by the early development of cognitive biases towards threat. Further research is required to explore other biases that may increase at-risk children’s vulnerability to developing anxiety disorders. Children of anxious parents are at serious risk of developing anxiety disorders themselves, and until we understand why this is the case, we have limited hope of breaking the cycle.
